# Detection of Dental Caries and Cracks with Quantitative Light-Induced Fluorescence in Comparison to Radiographic and Visual Examination: A Retrospective Case Study

**DOI:** 10.3390/s21051741

**Published:** 2021-03-03

**Authors:** Song Hee Oh, Sae Rom Lee, Jin Young Choi, Yong Suk Choi, Seong Hun Kim, Hong Cheol Yoon, Gerald Nelson

**Affiliations:** 1 Department of Oral and Maxillofacial Radiology, Graduate School, Kyung Hee University, Seoul 02447, Korea; Ohbbang50@gmail.com (S.H.O.); saerom0928@gmail.com (S.R.L.); omrcys@khu.ac.kr (Y.S.C.); 2 Department of Orthodontics, Graduate School, Kyung Hee University, Seoul 02447, Korea; joyful.ortho@gmail.com; 3 Private Practice, Bestden Dental Clinic, Seoul 06232, Korea; yhc@aiobio.co.kr; 4 Division of Orthodontics, Department of Orofacial Science, University of California, San Francisco, CA 94143, USA; gdnelson41@gmail.com

**Keywords:** dental caries, bitewing radiograph, quantitative light induced fluorescence, dental crack, diagnosis, X-ray

## Abstract

The aim of this study was to present an optimal diagnostic protocol by comparing and analyzing a conventional examination and the quantitative light-induced fluorescence (QLF) technique. Selected were 297 teeth of 153 patients to take QLF images and bitewing radiographs. Occlusal dental caries, proximal dental caries and cracks were evaluated and scored using QLF, X-ray and/or visual criteria. The sensitivity, specificity, and area under the curve (AUC) of a receiver operating characteristic analysis were calculated. Two fluorescence parameters (|ΔFmax| and ΔRmax) were utilized to evaluate the fluorescence pattern according to the severity of lesions based on QLF or X-ray criteria. QLF showed higher scores for detecting occlusal dental caries and cracks than the conventional method. ΔRmax increased more clearly than ΔFmax did with occlusal dental caries. The |ΔFmax| values of occlusal dental caries, proximal dental caries and cracks showed good AUC levels (0.84, 0.81 and 0.83, respectively). The ΔRmax of occlusal dental caries showed the highest AUC (0.91) and the ΔRmax of proximal dental caries showed a fail level (0.59) compared to bitewing radiographs. The QLF image could visualize and estimate the degree of occlusal dental caries or cracks. Consequently, the QLF technique may be an adjunct tool to conventional methods for the detection of occlusal caries and peripheral cracks.

## 1. Introduction

The prevalence of dental caries is on the decline worldwide due to the use of fluoride and increasing awareness of oral health. Nonetheless, the relative prevalence of non-cavitated lesions is rising [1,2]. To treat the initial stage of caries requires early detection, the evaluation of lesions, and non-operative preventive treatments, where accurate diagnosis is essential. The prevalence of occlusal dental caries missed on visual examination has been a clinical issue for several decades. Complex occlusal fissures can lead to misdiagnosis and mask the further development of undetected caries [3]. The detection and diagnosis of proximal caries is difficult because of limited visual access [4]. Tooth cracks are initially generated by the concentration of local stress on the enamel surface that can penetrate into the dentin as the load increases [5,6]. Tooth cracks tend to deteriorate into vertical root fractures and threaten the vitality of the tooth. However, detecting tooth cracks using conventional visual examination methods is diagnostically challenging [7]. Clinicians seek better methods for the detection and accurate diagnosis of carious lesions and dental cracks that are difficult to find in clinical examinations. Radiographic examination is a useful method to confirm a clinical suspicion of dental caries. Demineralized tissue is observed radiographically as the attenuation of X-rays is less than that in the normal tissue. In particular, bitewing radiography enables the early detection and diagnosis of early caries on the proximal surface [8,9]. However, determining the presence and extent of lesions on the occlusal and smooth surfaces with radiographic examination has limits. It is difficult to determine whether they are active or not. cone-beam computed tomographic (CBCT) imaging may be more accurate and useful than normal radiographs for diagnosing hard tissue lesions such as early caries or cracks [10]. However, the level of radiation exposure with CBCT imaging remains an issue to consider. Two-dimensional radiology already represents an acceptable diagnostic aid; the use of CBCT would not find any justification and would be in contrast with the “as low as reasonably achievable” (ALARA) principles. Recently, in order to overcome the limitations of traditional diagnostic methods, a method of caries detection using physical stimulation with lights and electrical currents has been developed. Those include methods such as quantitative light-induced fluorescence (QLF) and fiberoptic trans illumination (FOTI) using visual light, DIAGNOdent using laser light, and electrical conductance measurements (ECM) using an electrical current [11,12]. One representative method is QLF, which detects caries by quantifying the auto fluorescence emitted from teeth illuminated by light at 405 nm [13,14]. In addition, the QLF image can be used to assess the severity of caries by detecting red fluorescence from porphyrin, which is produced by oral bacteria and penetrates into the tooth surface, and to detect oral bacterial structures such as plaque and calculus [15,16,17]. It is possible to objectively quantify the lesion status using quantitative QLF parameters (e.g., ΔF, ΔQ, and ΔR) as calculated with existing software [18,19,20]. These devices are sensitive to external light, which can prevent a high-quality image. In proximal caries, the intensity of light transmitted through the occlusal surface is already reflected before actually reaching the lesion, so the detection is impossible if the degree of caries does not exceed a certain level [21]. There have been attempts to evaluate early lesions using such equipment, and research results have been reported on its validity. However, there are insufficient studies evaluating the reliability of this method compared to visual inspection and dental radiographs, which are clinically used for the diagnosis of early hard tissue lesions today. The purpose of this study was to find an optimal method for the clinical evaluation of early hard tissue lesions by comparing the reliability between the conventional examination and QLF examination methods.

## 2. Materials and Methods

**Figure 1:**
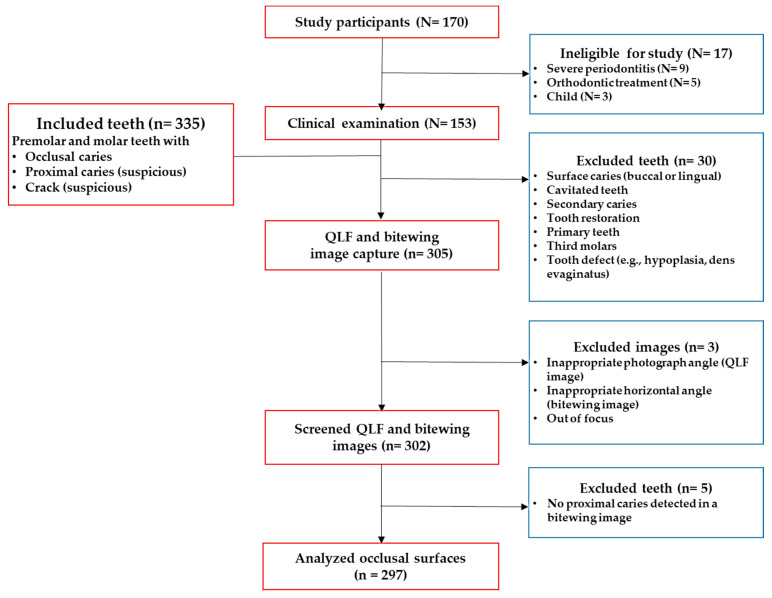
Flow diagram of the inclusion and exclusion for diagnosis of dental caries and tooth cracks (N = number of subjects, n = number of teeth); ICDAS = International Caries Detection and Assessment System [22]. QLF: quantitative light-induced fluorescence.

This study was approved by the Kyung Hee University Institutional Review Board (IRB No. KH-DT20032) followed by the tenets of the Declaration of Helsinki. Clinical data were collected throughout a clinical study performed from July to December 2020 at Kyung Hee University Dental Hospital, South Korea. The exclusion criteria were patients who had systematic diseases, had previous orthodontic treatments, had severe periodontitis or a Temporomandibular Joint (TMJ) disorder, based on pre-interview surveys. All subjects who visited Kyung Hee University Dental Healthcare Center of Kyung Hee University Dental hospital were given explanations regarding the objectives and procedures of this study. Those who subsequently provided written agreement to participate were over 18 years and in good health (N = 153) (Figure 1).

### 2.1. Clinical Examination

Along with a dental mirror, air syringe, and ball-type probe, the examiner conducted visual and physical examinations on the surfaces of premolar and molar teeth for the detection of occlusal caries, proximal caries and/or cracks. Surface (buccal or lingual) caries, cavitated teeth (ICDAS code = 6) [22], secondary occlusal caries, primary teeth, third molars, and teeth with hypoplasia or dens invaginatus that might affect the results were all excluded based on visual examinations. This procedure had a final inclusion of 297 teeth from 153 individuals (Figure 1).

### 2.2. QLF System

**Figure 2:**
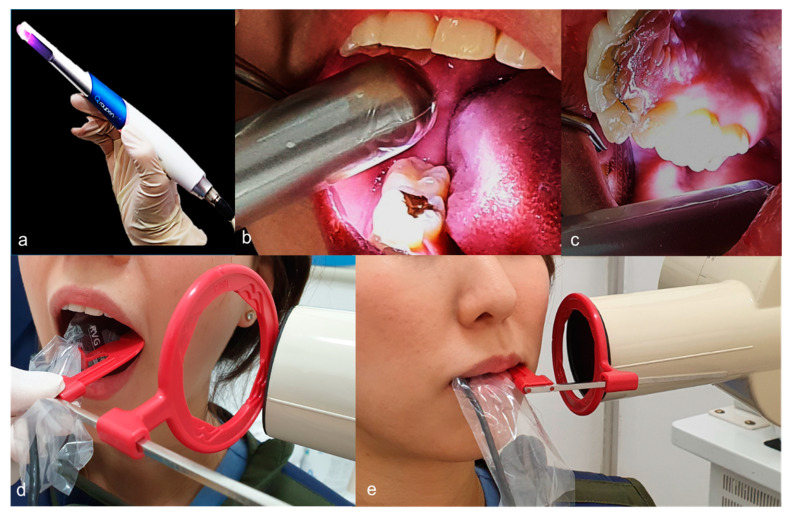
Diagnostic record taking procedure. (**a**–**c**) The quantitative light-induced fluorescence (QLF) images were obtained using a Qraypen C (AIOBIO, Seoul, Republic of Korea); (**d**,**e**) The standardized bite-wing radiographs were taken using digital sensor (Kodak RVG 6000, Carestream Dental, Rochester, NY, USA) and bitewing holder (XCP^®^ BAI Kit, Dentsply Rinn, York, PA, USA).

The QLF system is a device that can detect early dental caries by irradiating the teeth with visible light. The light used in QLF is 405 nm blue visible light. When this light is applied to healthy teeth, the light is transmitted to the dentino–enamel junction (DEJ) and then reflected, thereby generating green natural fluorescence. However, in an area of an early carious lesion, the light is scattered in the hard tissue of the lesion, and the fluorescence disappears and appears black. In addition, red fluorescence will appear from a substance named porphyrin, a metabolite secreted by bacteria in the oral cavity, enabling indirect evaluation of the progress of the lesion [23]. The white-light and fluorescence images were obtained by one examiner with a Qraypen C (AIOBIO, Seoul, Republic of Korea) in a dark room as to maintain and enhance the quality of the images (Figure 2a–c). Tooth cleaning was conducted with a toothbrush and gauze prior to taking the QLF image, as debris or plaque could affect results. Before taking the necessary images, the occlusal surfaces were dried sufficiently with compressed air. The device was positioned vertically over the occlusal surface. The images were automatically saved in bitmap format.

### 2.3. Bitewing Radiography

After taking QLF images, bitewing radiographs were taken for the same tooth. The standardized bitewing radiographs were taken using a digital sensor (Kodak RVG 6000, Carestream Dental, Rochester, NY, USA) and bitewing holder (XCP^®^ BAI Kit, Dentsply Rinn, York, PA, USA) (Figure 2d,e). A digital intraoral sensor on the lingual aspect of the tooth, and an X-ray unit (Asahi Roentgen Industry, Kyoto, Japan) was operated at exposure of 60 kV and 7 mA, with an average exposure time of 0.63 s. Images were simultaneously viewed on the monitor using the associated image program software (ZeTTA PACS, TaeYoung Soft, Anyang, Korea, http://taeyoungsoft.com/product01.php, accessed on 30 December 2020).

### 2.4. Scoring of QLF and Bitewing Images

The QLF score was calculated based on the QLF images. As previously reported, the score was calculated based on the following criteria [24,25]. The radiographic score was calculated based on the bitewing images.

The evaluation of the bitewing and QLF images was conducted by one specialist in oral and maxillofacial radiology with more than 10 years of experience. To prevent the QLF and bitewing images from affecting the evaluation, each evaluation sheet was prepared separately, and the bitewing and QLF images of the same patient on the same day were not evaluated at the same time. The criteria were applied conservatively, as lower grades were applied to lesions that were difficult to distinguish (Table 1).

### 2.5. Analysis of QLF Images Using QA2 Software

**Figure 3:**
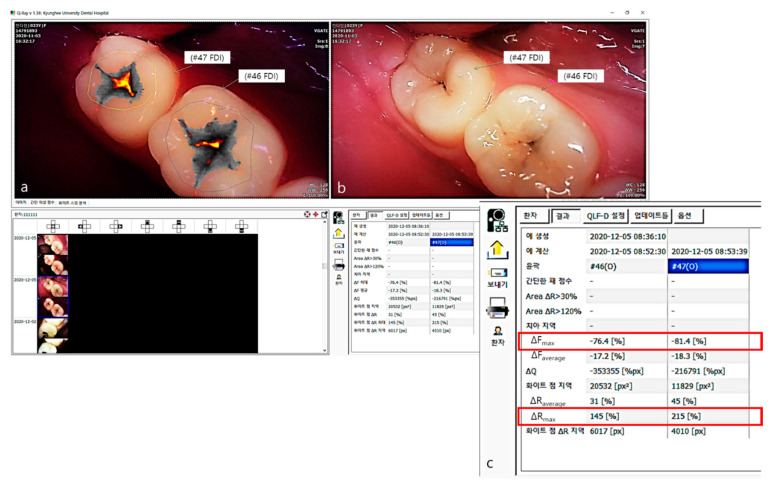
The QA2 program (version 1.25, Inspektor Research systems BV, Amsterdam, the Netherlands). (**a**) fluorescence image of QLF, the gray scale shows the degree of demineralization, and the yellow–orange color scale shows the intensity of red fluorescence by detecting from porphyrin.; (**b**) white-light image of QLF; (**c**) QA2 program provides fluorescence parameters (ΔFmax and ΔRmax) of occlusal surfaces.

All fluorescence images of the occlusal surface in this study were analyzed by one calibrated examiner. Factors such as staining and debris that may influence the evaluation of fluorescence images were confirmed via white-light images. Afterwards, the same examiner completed quantitative analyses in obtaining the fluorescence parameters of all occlusal surfaces using the QA2 program (Version 1.25, Inspektor Research systems BV, Amsterdam, The Netherlands) (Figure 3). For the fluorescence images, the study area was surrounded by sound enamel in accordance with manufacturer recommendations. The fluorescence changes in the occlusal surfaces were then calculated by the QA2 software algorithm, where it calculates the maximum loss of fluorescence (|ΔFmax|) and the maximum increase in red fluorescence (ΔRmax). The variable of |ΔFmax| was related to depth of lesion and mineral loss and ΔRmax indicates that porphyrin, a metabolite secreted by bacteria, is present in the oral cavity and assesses the level of bacterial activity.

### 2.6. Statistical Analysis

The intra-examiner reproducibility of the QLF score, bitewing score and QLF parameter was assessed in a second examination of 20 randomly selected teeth two weeks later, in which the intra-class correlation coefficient (ICC) value showed significant and excellent agreement (ICC > 0.9). To examine the correlation between the findings of the QLF score and bitewing score, the Spearman correlation coefficient was used; the distribution was confirmed using cross-tabulation. The mean values of QLF parameters were compared using one-way ANOVA and Tukey’s post-hoc analysis. Finally, a receiver operating characteristic (ROC) curve analysis was used to assess the sensitivity and specificity of the QLF parameters for dental caries or cracks, and to calculate the validity of an improved threshold value of the QLF parameters on clinical images using the area under the ROC curve (AUROC). The AUROC were calculated for occlusal dental caries for threshold of the QLF criteria: 0, 1 vs. 2, 3, proximal dental caries for threshold of the X-ray criteria: 0, 1 vs. 2–4 and crack for threshold of the QLF criteria: 0, 1 vs. 2. The significance cutoff for all statistical tests was set at α = 0.05 (version 23.0, PASW Statistics, SPSS, Chicago, IL, USA).

## 3. Results

In this study, a total of 297 teeth included the 177 teeth with occlusal dental caries, 91 teeth with proximal dental caries, and 29 teeth with cracks.

### 3.1. Occlusal Dental Caries

Classifying occlusal dental caries according to the QLF criteria and the X-ray criteria, most of the X-ray criteria results showed a value of 0, hence the correlation analysis between the two criteria was meaningless. On the other hand, the values of the QLF parameter (|ΔFmax|, ΔRmax) according to the value classified based on the QLF criteria increased significantly as the score value increased from 1 to 3 (from 50.67 to 77.42, from 49.34 to 221.87, respectively). In particular, ΔRmax was about 2.1-fold higher for the Occlusal dental caries score = 1 (105.65) than Occlusal dental caries score = 0 (49.34), and the red fluorescence intensity was 4.5-fold higher for Occlusal dental caries score = 2 (221.87) (Table 2).

The cut-off value of |ΔFmax| and ΔRmax for optimal sensitivity and specificity for determining occlusal dental caries (QLF criteria scores 0 and 1 vs. 2–4) was 59.85 and 74.50, respectively. In addition, the validity of the |ΔFmax| parameter for identifying occlusal dental caries was higher than the ΔRmax (Table 3).

**Figure 4:**
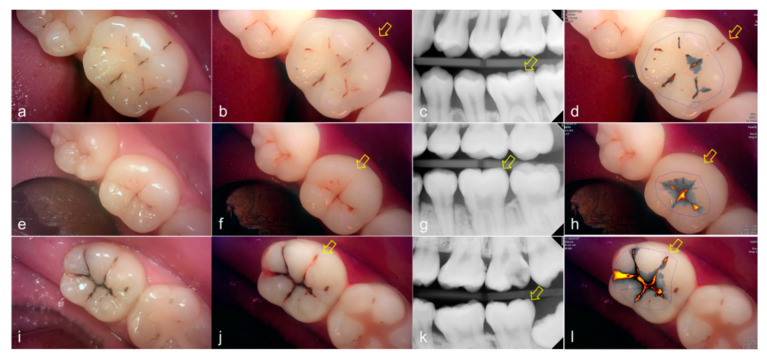
Occlusal dental caries: (**a**–**d**) QLF caries score 1 (fluorescence loss and red fluorescence present as a line or spot in pits and/or fissures) and radiographic caries score 0 (no radiolucency visible) on #36; (**e**–**h**) QLF caries score 2 (fluorescence loss and red fluorescence glow extending around pits and fissures) and radiographic caries score 0 (no radiolucency visible) on #36; (**i**–**l**) QLF caries score 3 (red fluorescence glow extending around pits and fissures and a dark shadow from dentin present) and radiographic caries score 0 (no radiolucency visible) on #37; (**a**,**e**,**i**): white-light image of QLF; (**b**,**f**,**j**): fluorescence image of QLF; (**c**,**g**,**k**): bitewing radiograph; (**d**,**h**,**l**): analyzed QLF image using QA2 software.

**Figure 5:**
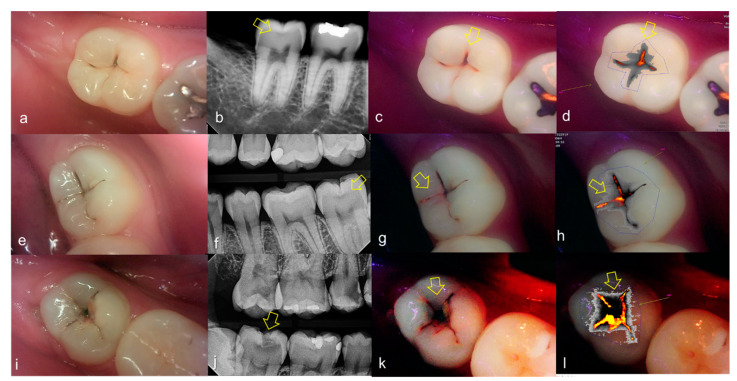
Occlusal dental caries: (**a**–**d**) QLF caries score 2 (fluorescence loss and red fluorescence glow extending around pits and fissures) and radiographic caries score 3 (radiolucency extending to the middle 1/3 of the dentine) on #47; (**e**–**h**) QLF caries score 3 (red fluorescence glow extending around pits and fissures and a dark shadow from dentin present) and radiographic caries score 3 (radiolucency extending to the middle 1/3 of the dentine) on #37; (**i**–**l**) QLF caries score 3 (red fluorescence glow extending around pits and fissures and a dark shadow from dentin present) and radiographic caries score 3 (radiolucency extending to the middle 1/3 of the dentine) on #47; (**a**,**e**,**i**): white-light image of QLF; (**b**,**f**,**j**): fluorescence image of QLF; (**c**,**g**,**k**): bitewing radiograph; (**d**,**h**,**l**): analyzed QLF image using QA2 software.

Representative images of QLF and bitewing radiograph of occlusal dental caries are shown in Figure 4 and Figure 5.

### 3.2. Proximal Dental Caries

**Figure 6:**
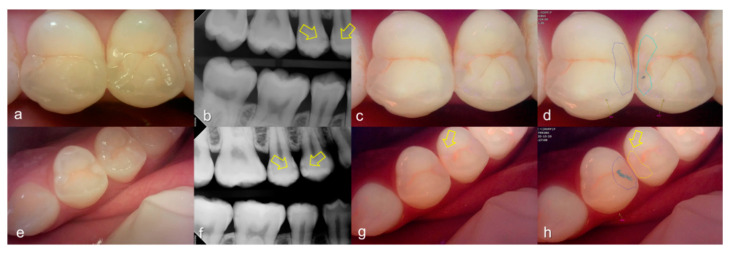
Proximal dental caries: (**a**–**d**) QLF caries score 0 (no fluorescence loss and no red fluorescence increase in occlusal surface (distal side)) and radiographic caries score 1 (radiolucency visible in the enamel) on #14 and QLF caries score 0 (no fluorescence loss and no red fluorescence increase in occlusal surface (mesial side)) and radiographic caries score 2 (radiolucency in the dentine but restricted to the outer 1/3 of the dentine) on #15; (**e**–**h**) QLF caries score 1 (fluorescence loss and red fluorescence present in occlusal surface (distal side)) and radiographic caries score 2 (radiolucency in the dentine but restricted to the outer 1/3 of the dentine) on #14 and QLF caries score 0 (no fluorescence loss and no red fluorescence increase in occlusal surface (distal side)) and radiographic caries score 2 (radiolucency in the dentine but restricted to the outer 1/3 of the dentine) on #15; (**a**,**e**): white-light image of QLF; (**b**,**f**): fluorescence image of QLF; (**c**,**g**): bitewing radiograph; (**d**,**h**): analyzed QLF image using QA2 software.

**Figure 7:**
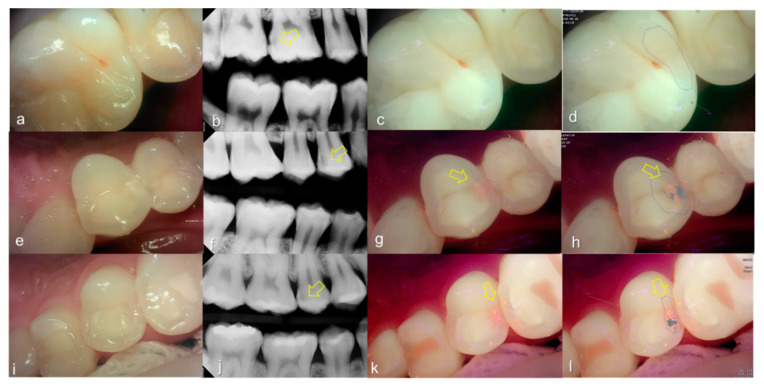
Proximal dental caries: (**a**–**d**) QLF caries score 0 (no fluorescence loss and no red fluorescence increase in occlusal surface (distal side)) and radiographic caries score 3 (radiolucency extending to the middle 1/3 of the dentine) on #16; (**e**–**h**) QLF caries score 2 (fluorescence loss and red fluorescence glow extending around occlusal surface (distal side)) and radiographic caries score 3 (radiolucency extending to the middle 1/3 of the dentine) on #14; (**i**–**l**) QLF caries score 2 (fluorescence loss and red fluorescence glow extending around occlusal surface (distal side)) and radiographic caries score 3 (radiolucency extending to the middle 1/3 of the dentine) on #15; (**a**,**e**,**i**): white-light image of QLF; (**b**,**f**,**j**): fluorescence image of QLF; (**c**,**g**,**k**): bitewing radiograph; (**d**,**h**,**l**): analyzed QLF image using QA2 software.

**Figure 8:**
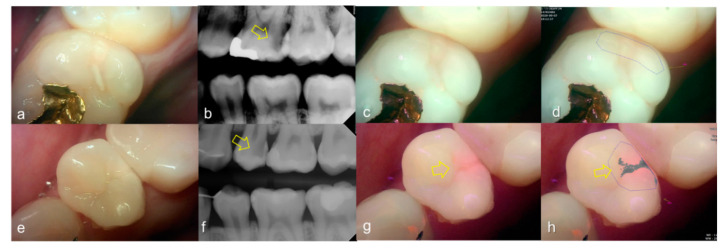
Proximal dental caries: (**a**–**d**) QLF caries score 0 (no fluorescence loss and no red fluorescence increase in occlusal surface (distal side)) and radiographic caries score 4 (radiolucency in the pulpal 1/3 of the dentine) on #26; (**e**–**h**) QLF caries score 2 (fluorescence loss and red fluorescence glow extending around occlusal surface (distal side)) and radiographic caries score 4 (radiolucency in the pulpal 1/3 of the dentine) on #25 (**a**,**e**): white-light image of QLF; (**b**,**f**): fluorescence image of QLF; (**c**,**g**): bitewing radiograph; (**d**,**h**): analyzed QLF image using QA2 software.

Evaluating proximal dental caries according to the QLF criteria and X-ray criteria, moderate positive correlation was observed (r = 0.63, *p* < 0.0001). Among the QLF parameters classified based on X-ray criteria, the |ΔFmax| value increased significantly as the score value increased from 1 to 4 (from 3.12 to 19.56). In particular, |ΔFmax| was about 5.7-fold higher for proximal dental caries score = 3 (17.64) than proximal dental caries score = 0 (3.12), and the maximum loss of fluorescence was 6.3-fold higher for the proximal dental caries score = 4 (19.56) (Table 2). The cut-off value of |ΔFmax| for detecting proximal dental caries (X-ray criteria scores 0 and 1 vs 2–4) was 5.95. The sensitivity, specificity and AUROC of parameter were 0.74, 0.73 and 0.81, respectively. However, the sensitivity, specificity and AUROC of the parameter ΔRmax were 0.83, 0.00 and 0.59 (Table 3). Representative images of QLF and bitewing radiograph of proximal dental caries are shown in Figure 6, Figure 7 and Figure 8.

### 3.3. Crack

**Figure 9:**
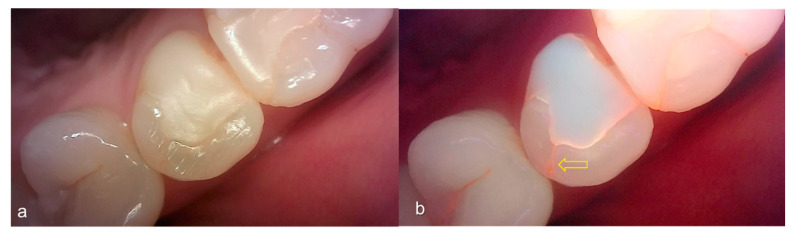
Cracks: (**a**) white-light image of QLF, visual examination score 0 (No detectable crack line) on #15; (**b**) fluorescence image of QLF, QLF caries score 1 (fluorescence loss and red fluorescence present as a line in a crack site) on #15.

**Figure 10:**
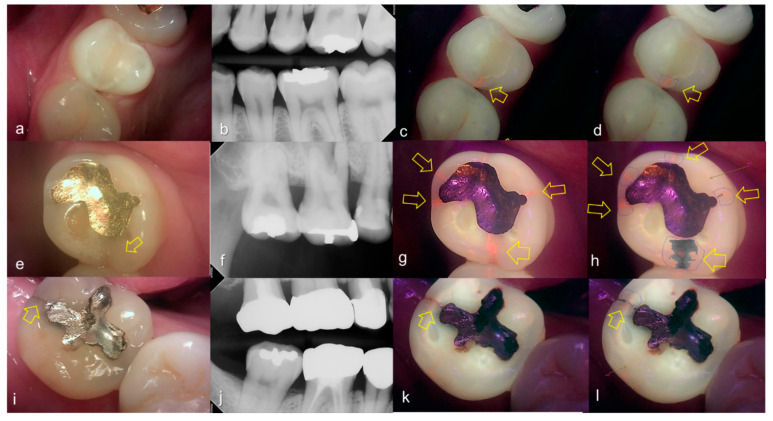
Cracks: (**a**–**d**) QLF caries score 2 (fluorescence loss and red fluorescence glow extending around crack site) and visual examination score 0 (no detectable crack line) on #25; (**e**–**h**) QLF caries score 2 (fluorescence loss and red fluorescence glow extending around a crack site) and visual examination score 1 (ambiguous detectable crack line) on #17 (arrow 1), QLF caries score 1 (fluorescence loss and red fluorescence present as a line in the crack site) and visual examination score 0 (no detectable crack line) on #17 (arrow 2); (**i**–**l**) QLF caries score 2 (fluorescence loss and red fluorescence glow extending around a crack site) and visual examination score 2 (distinct detectable crack line) on #47; (**a**,**e**,**i**): white-light image of QLF; (**b**,**f**,**j**): fluorescence image of QLF; (**c**,**g**,**k**): bitewing radiograph; (**d**,**h**,**l**): analyzed QLF image using QA2 software.

The values of the QLF parameter according to the value classified based on the QLF criteria were significantly different (Table 2). The cut-off value of |ΔFmax| and ΔRmax for determining a crack (QLF criteria scores 0 and 1 vs. 2) was 20.80 and 39.00, respectively. In addition, the AUROC of QLF parameters were similar (0.83, 0.82) (Table 3). Representative images of QLF and bitewing radiograph of dental cracks are shown in Figure 9 and Figure 10.

## 4. Discussion

For years, visual examinations combined with probing have been the key to diagnosing occlusal caries. It has also been shown that probing pressure has a potentiality of damaging fissures [27,28]. Unfortunately, however, visual assessment alone can leave numerous undetected dental caries in initial stages. Even though radiographs are known as commonly-used diagnostic methods, they still have limitations. Radiographs are only capable of detecting dental caries at a relatively advanced state, while quantitative assessments of the mineral changes occurring over time cannot be detected [29,30]. Furthermore, diagnosing early enamel caries is yet another problem, particularly in occlusal surfaces [31]. Accordingly, managing lesions of occlusal caries requires the precise and consistent detection of early non-cavitated lesions.

Recently, systems based on the QLF technology have been developed, which facilitates early non-cavitated caries detection and depth estimation [32]. The rationality, reproducibility and sensitivity of the QLF technique in detecting caries have formerly been assessed by other techniques such as chemical analysis, transverse microradiography, laser-induced fluorescence, longitudinal microradiography, [33,34] and optical coherence tomography [35]. QLF is capable of perceiving twice as many demineralized pre-cavitated enamel sites than visual examination or other caries detecting instruments [36].

Diagnosing proximal caries is challenging because of the anatomy of the lesions. It was concluded that 75% of proximal lesions are in the contact sites and the rest beneath them, which limits visual detection [37]. Proximal lesions are detected only when the marginal ridges become cavitated [38]. It is probable to underestimate the quantity of proximal caries with visual examination only. Although attempts have been made to diagnose proximal caries using the QLF technique, limitations have emerged due to the anatomical location of the lesion. At these sites, less fluorescence is produced while the lesion blocks the excitation light from the device and the back-scattered fluorescence from dentine. This results in the reduction of fluorescence, which makes it difficult to detect fluorescence in proximal dental caries [21]. However, another study showed that the QLF technology can be used as a screening tool to detect proximal dental caries at the dentine level prior to radiographic examination [39]. In this case, radiographic examination is another common method in recognizing proximal lesions, and bitewing radiographs can detect lesions at the early phase [8,9].

The main type of crack included in the current study was peripheral rim fractures. It mainly occurs around occlusal restorations. According to the peripheral rim theory, the preparation of cavities in the tooth structure disrupts the natural load distribution and creates zones of stress concentration. The clinical presentation of this stress concentration depends primarily on two factors: the type of cavity prepared, and whether the applied load is compressive or tensile [40].

One of the more challenging subjects regards cracked teeth. They are hard to visualize and to determine the appropriate treatment, given varying symptoms, depending on the direction and rate of progression [41,42]. Numerous crack-diagnosing methods have been used, such as methylene blue dye, microscopic examination, trans-illumination, bite test, radiography, cone-beam computed tomographic (CBCT) imaging, and optical coherence tomographic imaging [43,44,45,46]. Of these, visual inspection along with trans-illumination is reported to be more reliable in detecting tooth cracks [7]. However, this does not measure the depth of the cracks and then shows all cracks, including fine craze lines. CBCT imaging is more useful in detecting vertical fractures than periapical radiography. However, radiation exposure from CBCT imaging is a concern [47]. Patients are exposed to risk with the use of ionizing radiation, encouraging dental professionals to seek alternative methods.

QLF technology enables crack detection and depth assessment with no radiation [28]. It can calculate the depth by quantifying the loss of green fluorescence from back-scattering by decreased minerals. In previous studies, the maximum fluorescence loss increased with the depth of the crack, with a strong correlation between the depth itself and the |ΔFmax| value (correlation coefficient of 0.84, *p* < 0.01). It was found that |ΔFmax| is only affected by lesion’s deepest site. Thus, |ΔFmax| is considered to be a valid metric for quantifying enamel cracks [48]. Furthermore, for detecting bacterial deposits and bacteria-related lesions, red fluorescence from bacterial metabolites, such as porphyrin, is useful [49]. It is thought that a crack line with red fluorescence is older and more likely to have bacterial activity. Consequently, QLF can be used to detect enamel cracks and quantify their depth and age.

To evaluate the fluorescence patterns regarding the severity of lesions (such as dental caries and cracks) based on QLF or X-ray criteria, two fluorescence parameters (fluorescence loss and red fluorescence) were utilized. |ΔFmax| increased significantly with the lesion score (Table 2). Previous studies have established that the value of ΔF (reflecting the degree of mineral loss) is an index as it accurately reflects the depth of a lesion [50]. Particularly, a recent study confirmed that |ΔFmax| reflects the crack depth by measuring the maximum fluorescence loss [47]. Additionally, |ΔFmax| can be used to detect the fluorescence loss of a deep lesion on the occlusal surface [38,51]. Thus, fluorescence loss is suitable for evaluating the stage and depth of a lesion. Moreover, except for the proximal dental caries score, ΔRmax (another QLF parameter used in this study) can be used to calculate the depth of the lesion by considering its distinct differences compared to other scores. ΔRmax increased more drastically than ΔFmax did, especially in the occlusal dental caries, and so, changes in ΔRmax may be more suitable in distinguishing the depth of occlusal dental caries (Table 2). Previous studies, based on the results of histological analyses, showed that the intensity of red fluorescence increased as the lesion progressed and deepened [23]. Other studies have also shown that red fluorescence is more apparent in active lesions [16,52]. Although in this study, it was difficult to identify the relationship between the lesion’s activity and the presence of red fluorescence, if this is confirmed in the future, it will be possible to measure the caries status and provide appropriate treatments by evaluating red fluorescence level. Since the study was performed by a single trained examiner, the intra-examiner reliability was assessed. The ICC values showed all modalities with outstanding reproducibility. With this, however, it may be difficult to generalize the results. Therefore, further study with various examiners is necessary.

This study was performed to find an optimal method for evaluating early lesions in the clinical setting by comparing the reliability of the conventional methods and the QLF technique as a possible platform of the future dental healthcare system. In addition, a reliable dental diagnosis system based on this evaluation method will be developed and applied to various clinical situations. The objective and indirect diagnostic protocol through such images can be applied to the recently emerging telemedicine, which will make it more accessible in clinical practice [53].

## 5. Conclusions

In this study, conventional examination with visual inspection/bitewing radiographs and the quantitative light-induced fluorescence (QLF) techniques were evaluated and compared for the detection of occlusal dental caries, proximal dental caries and dental cracks. QLF showed a higher detection ability in detecting occlusal dental caries and cracks than the conventional method. Bitewing radiographs showed a higher rate for detecting proximal caries.

By combining of these complementary methods, it is believed that the number of missed lesions will be reduced and an accurate diagnosis of the initial lesion will be possible without unnecessary radiation. Further study with a larger sample could be implemented to verify this protocol.

## Figures and Tables

**Table 1:** Criteria of evaluation for dental caries and tooth crack.

	Score	ADA * Criteria	QLF Criteria	ADA * Criteria	X-ray Criteria
Occlusal dental caries	0	Sound	No fluorescence loss and no red fluorescence increase in pits and/or fissures	Sound	No radiolucency visible
	1	Initial	Fluorescence loss and red fluorescence present as a line or spot in pits and/or fissures	Initial	Radiolucency visible in the enamel
	2	Moderate	Fluorescence loss and red fluorescence glow extending around pits and fissures	Moderate	Radiolucency in the dentine but restricted to the outer 1/3 of the dentine
	3	Advanced	Red fluorescence glow extending around pits and fissures and a dark shadow from dentin present	Advanced	Radiolucency extending to the middle 1/3 of the dentine
Proximal dental caries	0	Sound or Initial	No fluorescence loss and no red fluorescence increase in occlusal surface (mesial or distal side)	Sound	
	1	Moderate	Fluorescence loss and red fluorescence present in occlusal surface (mesial or distal side)	Initial	Radiolucency visible in the enamel
	2	Advanced	Fluorescence loss and red fluorescence glow extending around occlusal surface (mesial or distal side)		Radiolucency in the dentine but restricted to the outer 1/3 of the dentine
	3			Moderate	Radiolucency extending to the middle 1/3 of the dentine
	4			Advanced	Radiolucency in the pulpal ^1^/_3_ of the dentine
	**Score**		**QLF Criteria**		**Visual Criteria**
Crack	0		No fluorescence loss and no red fluorescence increase in crack site		No detectable crack line
	1		Fluorescence loss and red fluorescence present as a line in crack site		Ambiguous detectable crack line
	2		Fluorescence loss and red fluorescence glow extending around crack site		Distinct detectable crack line

* Source: ADA (American Dental Association) Caries Classification System [26].

**Table 2:** Fluorescence parameters obtained from fluorescence images according to the severity of caries lesion classified using QLF or X-ray criteria.

Occlusal Dental Caries Score (QLF Criteria)	QLF Parameters			
	|ΔF_max_|	*p*-Value	ΔR_max_	*p*-Value
1	50.67 ^ab^	<0.0001 ^1^	49.34 ^a^	<0.0001 ^1^
2	63.69 ^ac^		105.65 ^b^	
3	77.42 ^bc^		221.87 ^ab^	
**Proximal Dental Caries Score** **(X-ray Criteria)**	**QLF Parameters**			
	**|ΔF_max_|**	***p*-Value**	**ΔR_max_**	***p*-Value**
1	3.12 ^ab^	<0.0001 ^1^	0.00	0.0083 ^1^
2	7.13 ^c^		0.00	
3	17.64 ^ac^		16.61	
4	19.56 ^b^		12.80	
**Crack Score** **(QLF Criteria)**	**QLF Parameters**			
	**|ΔF_max_|**	***p*-Value**	**ΔR_max_**	***p*-Value**
1	19.84	0.010 ^2^	22.44	0.011 ^2^
2	40.24		67.60	

^1^ Kruskal Wallis test, Bonferroni post hoc Differences between groups, marked with superscript lowercase letters in the same column are statistically significant. *p* < 0.05. ^2^ Mann–Whitney U test, *p* < 0.05.

**Table 3:** Cut-off value and validity of |ΔF_max|_ and ΔR_max_ for detecting dental caries and crack.

	Total Number of Teeth		Cut-Off Value	Sensitivity	Specificity	AUROC
Occlusal dental caries	177	|ΔF_max_|	59.85	0.76	0.74	0.84
		ΔR_max_	74.50	0.83	0.82	0.91
Proximal dental caries	91	|ΔF_max_|	5.95	0.74	0.73	0.81
		ΔR_max_	0.00	0.83	0.00	0.59
Crack	29	|ΔF_max_|	20.80	0.85	0.67	0.83
		ΔR_max_	39.00	0.75	0.78	0.82

## Data Availability

Not applicable.
